# Differences in condom access and use and associated factors between persons with and without disabilities receiving social cash transfers in Luapula province, Zambia—A cross-sectional study

**DOI:** 10.1371/journal.pone.0302182

**Published:** 2024-06-06

**Authors:** David Chipanta, Janne Estill, Heidi Stöckl, Elona Toska, Patrick Chanda, Jason Mwanza, Kelly Kaila, Chisangu Matome, Gelson Tembo, Olivia Keiser

**Affiliations:** 1 Institute of Global Health, University of Geneva, Geneva, Switzerland; 2 Institute for Medical Information Processing, Biometry, and Epidemiology, Medical Faculty, Ludwig-Maximilians-University Munich, Munich, Germany; 3 Centre for Social Science Research, University of Cape Town, Cape Town, South Africa; 4 Department of Social Policy and Intervention, University of Oxford, Oxford, United Kingdom; 5 Department of Sociology, University of Cape Town, Cape Town, South Africa; 6 Social Work and Sociology, University of Zambia, Lusaka, Zambia; 7 Disability Inclusion Project Luapula, International Labour Organisation, Lusaka, Zambia; 8 Palm Associates Limited, Lusaka, Zambia; 9 Economics and Agricultural Sciences, University of Zambia, Lusaka, Zambia; York St John University, UNITED KINGDOM

## Abstract

Persons with disabilities are disadvantaged in accessing sexual and reproductive health services, including condoms. In this study, we investigated whether condom access and use and their associated factors differed between persons with and without disabilities. We used data from adults in households receiving the Government of Zambia social cash transfers (SCT) in four districts of Luapula province. Condom access and use was the outcome. Disability, defined by the Washington Group Short Set Questions on Disability, was the main predictor. We performed logistic regression analyses to determine the associations between condom access and use and disability. In multivariable analyses, we controlled for covariates including age, sex, marital status, poverty status, HIV testing, and receiving the SCT. The sample comprised 1,143 people aged 16–49, with a median age of 21 years (interquartile range 18–28); 57.4% (n = 656) were female, 86.5% (n = 989) accessed and used condoms, and 17.9% (n = 205) were disabled, rating themselves with a 3 or a 4 on a scale of 1 = “not limited” to 4 = “cannot at all” in performing any of the six daily functions (seeing, hearing, walking, cognition, self-care, or communicating). Nearly sixty percent(58.5% (n = 120)) of persons with disabilities were female, 79.5% (n = 163) reported being very poor, 87.8% (n = 180) reported receiving SCT, and 86.3% (n = 177) reported accessing and using condoms. Condom access and use did not differ between persons with and without disabilities (adjusted odds ratio: 1.09; 95% confidence interval [CI]: 0.60–1.98]). We found no differences between persons with and without disabilities in condom access and use. We established that individual-level factors such as age, sex, marital status, and knowledge of being HIV positive might play a more important role in condom access and use than disability. Condom promotion interventions should account for these factors.

## Introduction

People’s access to and use of condoms is vital for preventing the transmission of the human immunodeficiency virus (HIV) and other sexually transmitted infections (STI). It is also crucial for meeting family planning needs. Globally, an estimated 1.7 million people newly acquired HIV in 2019 [1], and 376.4 million people acquired chlamydia, gonorrhea, syphilis, or trichomoniasis in 2016 [2]. Similarly, 190 million women of reproductive age had an unmet family planning need in 2019 [3]. By 2030, 10.9 billion male condoms (4.6 billion for family planning and 6.3 billion for HIV and STI prevention) will be required annually to avert the loss of 240 million disability-adjusted life years associated with HIV, STI and unplanned pregnancies [4]. However, persons with disabilities are disadvantaged, including in accessing sexual and reproductive health services [5]. Comprising 15% of the global population, persons with disabilities include those with long-term physical, mental, intellectual, or sensory conditions [5]. The interactions of these conditions with various barriers can hinder the full enjoyment of their rights [5]. Studies conducted among adults in sub-Saharan Africa, the region most affected by HIV, estimate HIV prevalence to be higher among persons with disabilities than in persons without [6–9]. These studies further show that women with disabilities are at a higher risk of acquiring HIV than women without [6–9]. Several studies also show that persons with disabilities, including female adolescents, are more likely to have sex with a casual partner without a condom and have a higher risk of acquiring an STI than their peers without disabilities [10–14]. The misperception that persons with disabilities are asexual is widespread. It may contribute to the disparity in their access to sexual and reproductive services [10, 13, 15]. This assumption prompted Zambia’s first person to declare his HIV-positive status, the late Winstone Zulu, who acquired polio in childhood, disabling both legs, to assert, "I had polio. I also have sex" [16]. Several factors influence persons with disabilities’ access to sexual and reproductive health services, including condoms [17]. These factors include age, gender, poverty, physical illness, proximity to health facilities, access to HIV testing, and participation in sexual and reproductive health training [18–23]. However, the extent to which condom access and use, and associated factors, differ between persons with and without disabilities is not known. The objective of this study was to investigate differences and associated factors in condom access and use between persons with and without disabilities. The research questions were: Did condom access and use differ between persons with and without disabilities? If so, did the differences persist by type and severity of the disability? What were the individual-level factors associated with condom access and use?

## Materials and methods

### Study design and sampling

We conducted this cross-sectional study in Kawambwa, Mansa, Nchelenge, and Samfya districts of Luapula province, located 760 kilometres north of Lusaka, Zambia’s capital. The district economy’s mainstay is agriculture, with fishing and trading constituting major livelihood activities [24].

We used data from poor households receiving the Government of Zambia Social Cash Transfers (SCT) in four districts of Luapula Province,, and participating in the United Nations Partnerships for the Rights of Persons with Disabilities (UNPRPD) Project. The UNPRPD implemented the project only in the Mansa and Samfya districts of Luapula province. To assess the UNPRPD project’s impact, we chose neighboring Kawambwa and Nchelenge districts as comparators. This selection helped to set up an impact evaluation, comparing outcomes between the project sites, Mansa and Samfya districts, and the non-project sites, Kawambwa and Nchelenge districts. We collected baseline data for the impact evaluation of the UNPRPD project from August to September 2019. The UNPRPD brings together UN entities, governments, organizations for persons with disabilities, and civil society to advance the rights of persons with disabilities around the world. In Zambia, the International Labour Organisation (ILO) implemented the UNPRPD project. The Ministry of Community Development and Social Services and other UN agencies supported the ILO in implementing the project.

The aim of the project was to increase access to sexual and reproductive health services for persons with disabilities receiving the SCT in Luapula province. Although the UNPRPD focused on persons with disabilities, the program was open to anyone receiving the SCT irrespective of disability status. Project interventions included training and outreach services, peer education programs, mass media campaigns, and capacity-building activities of public institutions, including health facilities and health worker training schools, community welfare assistance committees (CWACs), and persons with disabilities’ organisations on health and development domains. The planned evaluation aimed to assess the project’s impacts on several outcomes, including HIV testing and condom use. The project began in January 2019 and ended in December 2021. This study is based on the baseline data collected. We could not collect the endline data for the impact evaluation because of the COVID-19 pandemic restrictions.

We randomly sampled households receiving the SCT from CWACs. The CWACs are geographical areas designated by the Government of Zambia for administrative purposes. CWACs were the primary sampling units in this study. The CWACs are geographical areas designated by the Government of Zambia for administrative purposes in each district. CWACs consist of several groups of households, ranging from tens to hundreds. We calculated a minimum sample of 1800 households, comprising 90 clusters of CWACs, each with 20 households. We added five households, bringing the total to 25 households in each CWAC to account for households we would not locate. Our assumptions for the sample size calculations included an intervention effect of 0.20, a statistical significance of 0.05, a statistical power of 80% and Intra-class Correlation Coefficients (p) of 0.01–0.08 based on similar studies. First, for each district, we sampled CWACs using weights proportional to the number of households. Then, from each sampled CWAC, we sampled 25 households. Then from the household, we interviewed the head of household and adults aged 16 years and older. The University of Zambia Humanities and Social Sciences Research Ethics Committee (IRB Approval No. 2019-April-001) and the ethics committee of the Canton of Geneva (no 2019–00500) reviewed and approved the study protocol.

### Study population

We drew the sample from extremely poor households receiving the SCT. Households, are defined as units of people who live together in the same house. Households were eligible to receive the SCT if government authorities identified them as extremely poor by standards of living measures and if they satisfied one or more of the following criteria: being women-headed; being headed by a person aged 65 years or older; having a household member with a visual, physical, or sensory disability as certified by a government medical doctor; or having adults of working age who were unable to work or economically support themselves.

### Procedures

A questionnaire was administered face-to-face by trained data collectors, in the local Bemba language, separately to the head-of-household and each household member aged 16 or older who consented to participate in the study; 16 is the age of consent and suffrage in Zambia. Each respondent provided informed verbal and written consent, or thumb prints if they could not write that were recorded on the electronic tablets used for the data collection. Participation in the research was voluntary and did not affect the respondents’ receipt of the SCT.

The survey included questions on the respondents’ social demographics, proximity to health care facilities, and access to and use of a range of services such as condoms, HIV testing and receiving test results, sexual and reproductive health services, mobile phones, and social protection. We also asked about respondents’ disability status, experiences of physical and sexual gender-based violence, and physical locations including village and district. We drew the questions from piloted and validated tools including the UNICEF (United Nations Children’s Emergency Fund) Innocenti Survey Tools, UNAIDS HIV and Social Protection Assessment Tool, and the Demographic and Health Survey (DHS) [10, 25–29]. See S2 Annex for the data collection instrument in English and Bemba.

When training the data collectors, we translated the questions into Bemba from English and role-played the administration of the questionnaire to ensure a standardized understanding of the questions. We piloted the survey tool in Lusaka in an area socioeconomically comparable with the research area and found that the questions were standardized and easy to administer and understand. We did not change the questions after the pilot.

The interview at the respondent’s home or another place of their choice lasted 45 minutes. Data collectors were matched as much as possible to the respondents according to age and gender to mitigate bias in responses. A supervisor led each team of data collectors and accompanied the data collectors during the interviews. The supervisors were responsible for data quality. The supervisor reviewed a sample of responses from their team to ensure complete responses. They requested that the data collectors return to the households to complete the questionnaire in case of incomplete responses without a justifiable reason. The data collectors automatically recorded each household’s geographical position of each household on the electronic tablet. We did not reward the respondents financially for participating in the survey but gave them reference numbers to follow-up on accessing specific services or more information about the research. The data were transcribed on the electronic tablets with Open Data Kit software and transferred to and stored electronically on a secure server.

### Statistical analyses

#### Measures

The primary outcome variable was access and use of male and female condoms, operationalized with this binary (yes/no) question from the DHS: “If you want to use a condom (male and female), would it be easy to get or use?” [29]. We measured disability, the main predictor, with the Washington Group Short Set Questions on Disability (WGSS) [27]. The WGSS asked if people have difficulties in any of the six functional domains: seeing, hearing, walking, cognition, self-care, or communicating. For each disability type, the answer options are the following: 1, “no”; 2 “yes—a little”; 3 “yes—a lot”; 4 “cannot at all” (S1 Annex). We defined a respondent as disabled if they answered 3 or 4 for any domain.

Because the WGSS assesses functional difficulties including disabilities across six domains, it captures multiple disabilities because a person can give themselves a 3 or 4 rating in multiple domains. Notably, a person medically classified as disabled should be unlikely to rate themselves at 2 or 1 because medical certification measures total restriction in functioning.

Additional individual-level sociodemographic variables included age in years (16–24, 25–34, 35–49), sex (male, female), marital status (not married, married/having a partner), and HIV testing and receipt of result results (not tested, tested and received a negative result, tested and received a positive result) as shown in Table 1.

**Table 1:** Variable description and measurement scales.

Variable name	Question	Measurement
**Age (Years)**	What is your age in years?	Categorical:16–24, 25–34, 35–49
**Gender**	What is your gender?	Binary: male, female
**Marital status**	What is your marital status?	Binary: not married, married
**No poverty**	Do you consider your household to be not poor, moderately poor, or very poor?	Binary: very poor, moderately or not poor
**Distance to Health Facility (kilometres)**	Do you know where the nearest health facility (health post/centre/clinic/hospital) is? How far is it located in kilometres?	Categorical: Don’t know, 1 to 7 km, 8 km or more
**Access and use of condoms**	If you want condoms (male and female), would they be easy to get or use?	Binary: no, yes
**HIV testing and results**	Have you ever been tested for HIV? If yes, what was the result of your most recent test?	Categorical: not tested, tested and received negative result, tested and received positive results
**Government—Social Cash Transfer**	During the past 12 months, have you or any of the household members received money or goods, including food, clothing, livestock, or medicines from any of the following government programs?	Binary: no, yes

The existing evidence and our knowledge of the field guided the variable selection. De Beaudrap and colleagues, in their systematic review and studies in Cameroon on HIV disability, found HIV was higher among persons with than persons without disability (7–10). They found that women with disability were especially affected and that their experience of intimate partner violence elevated their risk of HIV (7–10). De Beaudrap et al. concluded that social and economic factors such as education level shaped women with disability’s vulnerability to HIV and impacted their access to sexual and reproductive health, including condoms (7–10,33).

Other studies examined factors associated with condom use among persons with physical disability in an urban town of Cameroon, university students and people living with HIV in Uganda, and people living with HIV in Brazil. They found that several factors impacted condom use (19–23). For example, among people living with HIV in Uganda, having a secondary or high level of education increased condom use (23). Being single and not disclosing HIV-positive status increased condom use among people living with HIV in Brazil (22). In Cameroon, the perceptions of the severity of HIV infection, benefits of condom use, and personal efficacy of using condoms were associated with increased condom use among persons with disabilities (19). However, none of these studies was from among people identified as extremely poor and receiving SCT.

SCT recipients are a vulnerable population facing increased attention in policy circles in expanding and improving the integrated delivery of the cash transfers with sexual and reproductive health services. Our study adds evidence to improve understanding of the specific factors influencing access to and use of condoms among persons with and without disability receiving SCT to support strengthened planning and delivery of the SCTs and meeting the sexual and reproductive needs of persons with and without disability living in poverty.

#### Analysis

We used manual stepwise variable selection in developing the model. Starting with the multivariable model, which included proximity to market, public transportation, food insecurity, a proxy for STI, and intimate partner violence, we removed variables with p values greater than 0.1 (p > 0.1) and then added them back individually, removing those with p > 0.1 and retaining those with p < 0.1, until the model contained only important variables identified by the likelihood ratio tests between the initial and reduced model. In controlling for multicollinearity and correlation, variables were removed or added to the model until the variance inflation factor was less than five and correlation less than 0.4. The final model included disability, age, marital status, sex, poverty level, distance to the nearest health facility, HIV testing, receipt of HIV test results, and receiving SCT. Condom access and use was the dependent variable.

Missing values were imputed using multiple imputation by chained equations, Stata 14.1 package, for variables with at least 5% missing; in addition to the variables in the model, we included mobile phone access, district, and sex of the head-of-household to improve the imputation of the missing data [30], and we included the outcome variable in the imputation. We ran the models on ten imputed data sets and performed univariable and multivariable logistic regression analyses to determine the association between the outcome variable and disability. In the multivariable analysis, we controlled for age, marital status, sex, poverty level, distance to the nearest health facility, HIV testing, receipt of HIV test results, and receiving SCT.

We clustered the analyses at the CWAC level using the cluster (, cluster ()) option command in Stata/SE 14.1. We presented the results of the final model as odds ratios with 95% confidence intervals (CI). The analyses were also stratified by sex, type of disability, and a rating of “a little difficult” in any functional domain. We further assigned difficulty levels to each of the six domains to create a functional severity score: 0 for no difficulty, 1 for some difficulty, 6 for a lot of difficulty, and 36 for cannot at all [31] to capture multiple disabilities and severity of functional difficulties.

## Results

The sample comprised 1,143 respondents aged 16–49 years. More than half (n = 656, 57.4%) were female. The median age was 21 years (interquartile range 18–28). A vast majority rated themselves very poor, reported living within seven kilometers of the nearest health facility, receiving the SCT and accessing and using condoms. Nearly one in five (17.9%;n = 205) had a disability, that is they answered 3 (“limited a lot”) or 4 (“cannot at all”) in performing any of the following: seeing, hearing, walking, cognition, self-care, or communicating (Table 2). Although 58.8% (n = 671) had no functional difficulty, 23.3% (n = 266) had mild, 16.7% (n = 191) moderate and 1.2% (n = 14) had severe difficulty. Nine percent (103/1143) of the condom access and use variable data were missing (and was imputed).

**Table 2:** Characteristics of respondents by disability status.

Variables	Not Disabled		Disabled		*p value*	Total	
	n	%	n	%		n	%
**Age in Years**							
16–24	640	68.2	117	57.1		757	66.2
25–34	171	18.2	38	18.5		209	18.3
35–49	127	13,5	50	24,4	*0*.*001*	177	15,5
**Gender**							
Male	402	42.9	85	41.5		487	42.6
Female	536	57.1	120	58,5	*0*.*388*	656	57.4
**Marital Status**							
Singles	803	85.6	169	82.4		972	85.0
Paired	135	14.4	36	17,6	*0*.*148*	171	15.0
**No Poverty**							
Very Poor	651	69.4	163	79,5		814	71.2
Moderately Poor	287	30.6	42	20.5	*0*.*002*	329	28.8
**Distance to Health Facility (Kilometres)**							
Don’t know	30	3.2	5	2.4		35	3.1
0 to 7km	780	83.2	174	84.9		954	83.5
8 or more	128	13.6	26	12.7	*0*.*839*	154	13.5
**HIV Testing and Results**							
Not tested	220	23.5	51	24.9		271	23.7
Negative	681	72.6	143	69.8		824	72.1
Positive	34	3.6	11	5.4		45	3.9
Missing	3	0.3	0	0.0	*0*.*399*	3	0.3
**Social Cash Transfer**							
No	79	8.4	25	12.2		104	9.1
Yes	859	91.6	180	87.8	*0*.*062*	1039	90.9
**Condom access and use**							
No	126	13.4	28	13.7		154	13.5
Yes	812	86.6	177	86.3	*0*.*504*	989	86.5

Fisher’s exact test. Disability was defined as answering 3 (“limited a lot”) or 4 (“cannot at all”) in performing any of the six functions.

Most characteristics of persons with disabilities were not different from persons without disabilities. However, Persons with disabilities tended to be older, and they rated themselves more frequently very poor than did persons without disabilities (Table 2).

Table 3 displays unadjusted and adjusted differences in factors associated with self-reported access to and use of condoms between persons with and without disabilities. There were no associations between condom access and use and disability before adjustment (unadjusted odds ratio 0.94, 95% CI: 0.55–1.59). No differences emerged between persons with and without disabilities in the access to and use of condoms after adjusting for age, sex, marital status, poverty level, distance to the nearest health facility, HIV testing and receipt of HIV test results, and receiving SCT.

**Table 3:** Factors associated with condom access and use (odds ratios, 95% CI).

Variables	Unadjusted n = 1143)	*p value*	Adjusted n = 1140	*P value*
**Disability**				
Not disabled	Ref		Ref	
Disabled	0.94[0.55–1.59]	*0*.*801*	1.09[0.60–1.98]	*0*.*757*
**Age in Years**				
16–24	Ref		Ref	
25–34	0.77[0.48–1.22]		0.84[0.52–1.36]	
35–49	0.38[0.22–0.64]	*<0*.*001*	0.59[0.31–1.16]	*0*.*149*
**Gender**				
Male	Ref		Ref	
Female	0.65[0.47–0.90]	*0*.*011*	0.52[0.36–0.75]	*<0*.*001*
**Marital Status**				
Singles	Ref		Ref	
Paired	0.29[0.19–0.45]	*<0*.*001*	0.31[0.18–0.53]	*<0*.*001*
**No Poverty**				
Very Poor	Ref		Ref	
Moderately Poor	0.93[0.60–1.45]	*0*.*759*	0.99[0.63–1.56]	*0*.*946*
**Distance to Health Facility (Kilometres)**				
0 to 7km	Ref		Ref	
Don’t know	0.77[0.23–2.56]		0.75[0.20–2.73]	
8 or more	1.49[0.76–2.92]	*0*.*437*	1.60[0.81–3.15]	*0*.*163*
**HIV Testing and Results** *				
Negative	Ref		Ref	
Not tested	1.00[0.60–1.65]		0.63[0.38–1.05]	
Positive	3.04[0.70–13.17]	*0*.*398*	4.30[0.95–19.40]	*0*.*005*
**Social Cash Transfer Access**				
No	Ref		Ref	
Yes	1.82[0.90–3.69]	*0*.*093*	1.69[0.81–3.51]	*0*.*135*

Wald test p value, ref = reference,

*n = 1140, Variable was not imputed because only three values were missing. Disability was defined as answering 3 (“limited a lot”) or 4 (“cannot at all”) in performing any of the six functions.

Compared with 16- to 24-year-olds, persons aged 35 to 49 years were less likely to access and use condoms before adjustment. However, these differences disappeared after adjustment. Similarly, in the unadjusted model, women had 35% lower odds of condom access and use than men, worsening to 48% after adjustment. Married persons or couples had nearly 70% lower odds of accessing and using condoms than singles before and after adjustment. (Table 3).

The results of the stratified analyses including "a little difficulty” in any functional domains in the definition of disability, by women and men separately, type of disability, or severity of functional difficulties were consistent with those in the adjusted analyses that defined disability as activity difficulty ratings of “a lot” or “cannot do at all” (S1–S3 Tables).

## Discussion

For this study, we examined the differences in access and use of condoms and associated factors between persons with and without disabilities. We found no differences in condom access and use between persons with and without disabilities disability type, or severity of functional difficulty or gender. The secondary results, which are consistent with a large body of evidence, were that the odds of accessing and using condoms were lower for women than men and for partnered respondents than singles but higher among persons with HIV-positive test results than persons with negative results.

Our primary finding of no differences in condom access and use between persons with and without disabilities contradicts a growing body of evidence that condom access and use are one of several areas in which persons with disabilities are disadvantaged. For example, authors of a study in Cameroon found that persons with disabilities were less likely to have used any modern family planning methods than were persons without disabilities [32]. Additionally, in a multicountry study involving Burkina Faso, Cape Verde, Guinea, and Niger, persons with disabilities were less likely to access condoms, women with disabilities faced more difficulties in accessing condoms than men [13], and similar results were obtained in Uganda [10].

In other studies, investigators observed differences in condom use by disability type and severity. In a cross-sectional comparative study in Nigeria, persons with mild or moderate intellectual disability were significantly more likely to have reported inconsistent or no condom use than were their peers without disabilities [33]. In another cross-sectional study, of heterosexual adults with low socioeconomic status in 17 states of the United States, found that persons with disabilities were more likely to have condomless sex than were persons without disabilities. The disabilities included hearing, seeing, walking, cognition, self-care, and difficulties with independent living [14].

One possible explanation of our null finding on differences in condom access and use is that persons with disabilities were like those without disabilities in our study in characteristics except for age and self-rated poverty. For instance, in our study persons with disabilities reported condom access and use, having tested for HIV, living within 7 km of the nearest health facility, and receiving SCT as often as those without disabilities. Another reason is that our sample comprised people experiencing poverty and deprivation independent of disability, and multidimensional poverty has been found to be associated with lower access to health services, including sexual and reproductive health, and condoms [32, 34, 35].

Although 17.9% of participants had a disability and a higher percentage of those disabled rated themselves "very poor" than did those without a disability, both groups experienced multidimensional poverty as a requirement for participating in the SCT. As in other studies, the high level of multidimensional poverty by both groups in our study might have accounted for the lack of differences in condom access and use between the two groups [32]. This result suggests that interventions to increase condom access and use among persons living in poverty should be universal to all including persons with disabilities. For disabled persons, these services should be delivered in their physical and social contexts, in their communities and the places they frequent such as health facilities or disabled persons organizations. In the case of SCT recipients, condom promotion could be integrated into the disbursement of cash transfers at the collection point or electronic payments could be bundled with information on condoms.

Our finding that women were less likely to access and use condoms is consistent with prevailing evidence. In a study in Cameroon, women with disabilities faced more difficulties accessing and using condoms than men, particularly those with no secondary school education [32]. Women have on average lower education than men, and the differences in education are worse for women with disabilities [32, 34]. Lower education levels might be reflected in less knowledge on condom access and use. For example, women with disabilities have been found to have more knowledge gaps in the correct use of condoms and in the role of condoms as a critical prevention method than their peers without disabilities [8, 18, 36]. Interventions to increase condom access and use among women, such as economic empowerment and increasing their negotiation skills (in addition to working to change male gender norms regarding condom use) should include specific activities focusing on women with disabilities including reducing intimate partner violence.

Another finding from our study was that being partnered or married was associated with lower condom access and use than was being single, which supports earlier evidence of lower condom access and use among married/partnered people than singles [37]. Men and women in a long-term relationship may not have the power, autonomy, and agency to negotiate the use of condoms. Additionally, the initiation of condom access and use among married couples and in other stable partnerships may signal a lack of trust [38].

The result of higher condom access and use among people who tested positive for HIV than among those who tested negative is supported by evidence from previous studies [39, 40]. In our study, respondents who had received an HIV test and positive results had fourfold higher odds of condom access and use than did peers who tested negative, although the differences in condom access and use were not apparent in the unadjusted model. They emerged after we adjusted for several individual-level factors, suggesting that HIV-positive test results combined with individual-level factors may have motivated condom access and use, for women.

Our results should be interpreted cautiously. We operationalized access and use of condoms by asking the DHS question of whether condoms would be easy to acquire and use if one desired, which was a simple yes/no question, so we could not distinguish between access and use of condoms. Further, because of social desirability bias, respondents might have overstated their ability to access and use condoms; the data collectors were unknown to the respondents, mitigating but not eliminating the social desirability bias. Additionally, the responses were self-reports, but self-reports of increased access to and use of condoms might not have translated to practical condom access and use skills.

These results are also not representative of the general population; they are restricted to adults who were living in extremely poor households receiving SCT, who tended to be unmarried, and there was a higher proportion of persons with disabilities than in the general population. This latter finding reflected two of the SCT eligibility criteria, being a female-headed household (which tend to be poorer) and having a disability. However, one in ten people reported not receiving SCT in the last 12 months, and not having received the transfer (i.e., not have cash) might have affected their access and use of condoms; we could not examine the impact of not receiving the transfers on condom access and use because of smaller sample size. Efforts are required to make transfers to all SCT recipients.

Further, the WGSS does not cover all disabilities and is also not good at capturing responses from persons with disabilities in cognition. We did not control for education level and employment status, which both affect condom access and use [32], because data on these variables were not available. We did not control for the fertility desires of respondents in our study, and some condom nonusers might have intended to be pregnant. We also could not know individuals’ numbers of sex partners, the agreement or discordance in partners’ HIV test results, or their knowledge of each other’s HIV status known, all of which could be associated with condom access and use (19–24). None of the respondents reported being non-poor. Since the sample was based on poor people, non-poor respondents might have been afraid to report that they were non-poor lest they lose the SCT. Lastly, the imputation may have affected the robustness of the results.

One main strength of this study is that we included multiple individual-level social economic variables, shining light on individual-level factors associated with condom access and use among people with and without disability living in poverty. Another strength is that we used data from a typical SCT program implemented in a lower- and middle-income setting, providing evidence that could be generalizable to similar SCT programs in sub-Saharan Africa. Our study is one of the first to find that among people living in poverty, condom access and use do not necessarily differ between persons with and without disabilities using cross-sectional data. Research including an impact evaluation of the UNPRPD is required to understand the types of interventions that can increase access to condoms for persons with disabilities. However, the full range of HIV prevention and family planning options and services, including condoms, should be universally available to everyone including persons with disabilities [41, 42] as human rights imperatives for preventing HIV and STI and meeting family planning needs.

## Conclusions

In this study, we found no evidence for differences in condom access and use for HIV/STI prevention and family planning between persons with and without disabilities. However, we established that multiple deprivations and individual-level factors such as gender, marital status, and testing HIV positive might play a more important role in condom access and use than disability. Condom promotion interventions should account for these factors. More research among people receiving SCT is required for understanding the differences between persons with and without disability in their condom access and use and associated factors.

## Supporting information

S1 Table Differences in factors associated with condoms access and use by gender (adjusted), odds ratios, 95% CI, p value. (DOCX)

S2 Table Differences in factors associated with condoms access and use by disability type (adjusted) odds ratios, 95% CI, p values. (DOCX)

S3 Table Differences in factors associated with condoms access and use among 16–49 year olds, odds ratios, 95% CI. (DOCX)

S1 Annex The Washington group short set of questions on disability. (DOCX)

S2 Annex Impact of social protection on access and use of HIV services: A quasi experiment study in two urban and two rural districts of Zambia household questionnaire. (DOCX)

S3 Annex Impact of social protection on access and use of HIV services: A quasi experiment study in two urban and two rural districts of Zambia household questionnaire (Bemba). (DOCX)
