# Supplementary material for: Differences in condom access and use and associated factors between persons with and without disabilities receiving social cash transfers in Luapula province, Zambia—A cross-sectional study
Source: PLoS One. 2024 Jun 6;19(6):e0302182. doi: 10.1371/journal.pone.0302182 (PMC11156379; doi:10.1371/journal.pone.0302182)
Supplement: S1 Table — (DOCX) [file pone.0302182.s001.docx]

| **S1 Table 1: Differences in factors associated with condoms access and use by gender (adjusted), Odds Ratios, 95% CI, p value** | | | | | | | | |
| --- | --- | --- | --- | --- | --- | --- | --- | --- |
| **Variables** | **Male (n=487)** | |  | **Female (n=653)** | |  | **Any disability^a^ (n=1126)** | |
| **Disability** |  |  |  |  |  |  |  |  |
| Not Disabled | Ref |  |  | Ref |  |  | Ref |  |
| Disabled | 0.76[0.31 - 1.90] | *0.577* |  | 1.25[0.59 - 2.62] | *0.543* |  | 0.85[0.56 - 1.29] | *0.443* |
|  |  |  |  |  |  |  |  |  |
| **Age in Years** |  |  |  |  |  |  |  |  |
| 16 - 24 | Ref |  |  | Ref |  |  | Ref |  |
| 25 - 34 | 1.24[0.42 - 3.68] | |  | 0.77[0.45 - 1.34] |  |  | 0.89[0.53 - 1.50] |  |
| 35 - 49 | 1.25[0.43 - 3.62] | *0.675* |  | 0.49[0.22 - 1.13] | *0.114* |  | 0.60[0.30 - 1.20] | *0.196* |
|  |  |  |  |  |  |  |  |  |
| **Sex** |  |  |  |  |  |  |  |  |
| Male |  |  |  |  |  |  | Ref |  |
| Female |  |  |  |  |  |  | 0.54[0.36 - 0.81] | *0.002* |
|  |  |  |  |  |  |  |  |  |
| **Marital Status** |  |  |  |  |  |  |  |  |
| Single | Ref |  |  | Ref |  |  | Ref |  |
| Paired | 0.10[0.04 - 0.29] | *<0.001* |  | 0.50[0.276- 0.96] | *0.025* |  | 0.30[0.17 - 0.52] | *0.000* |
|  |  |  |  |  |  |  |  |  |
| **No Poverty** |  |  |  |  |  |  |  |  |
| Very Poor | Ref |  |  | Ref |  |  | Ref |  |
| Moderately Poor | 1.18[0.52 - 2.70] | *0.684* |  | 0.88[0.51 - 1.52] | *0.627* |  | 0.93[0.62 - 1.40] | *0.724* |
|  |  |  |  |  |  |  |  |  |
| **Distance to Health Facility (Kilometres)** | | |  |  |  |  |  |  |
| 0 to 7km | Ref |  |  | Ref |  |  | Ref |  |
| Don’t know | 0.51[0.12 - 2.19] | |  | 1.15[0.20 - 6.43] |  |  | 1.04[0.26 - 4.11] |  |
| 8 or more | 2.80[0.63 - 12.33] | *0.151* |  | 1.29[0.59 - 2.82] | *0.542* |  | 1.51[0.33 - 6.95] | *0.265* |
|  |  |  |  |  |  |  |  |  |
| **HIV Testing and Results** | |  |  |  |  |  |  |  |
| Negative | Ref |  |  | Ref |  |  | Ref |  |
| Not tested | 0.65[0.26 - 1.62] |  |  | 0.58[0.30 - 1.10] |  |  | 1.40[0.89 - 2.19] |  |
| Positive | 3.25[0.38 - 28.19] | *0.170* |  | 5.52[0.68 - 45.01] | *0.007* |  | 6.16[1.30 - 29.14] | *0.008* |
|  |  |  |  |  |  |  |  |  |
| **Social Cash Transfer Access** | |  |  |  |  |  |  |  |
| No | Ref |  |  | Ref |  |  | Ref |  |
| Yes | 1.66[0.62 - 4.46] | *0.216* |  | 1.54[0.62 - 3.82] | *0.343* |  | 1.55[0.81 - 2.99] | *0.188* |
| Wald Test, ref = reference. ^a^Disability was defined as "a little difficulty" in performing any of six functions. | | | | | | | | |
