# Supplementary material for: Differences in condom access and use and associated factors between persons with and without disabilities receiving social cash transfers in Luapula province, Zambia—A cross-sectional study
Source: PLoS One. 2024 Jun 6;19(6):e0302182. doi: 10.1371/journal.pone.0302182 (PMC11156379; doi:10.1371/journal.pone.0302182)
Supplement: S2 Table — (DOCX) [file pone.0302182.s002.docx]

| **S1 Table 2: Differences in factors associated with condoms access and use by disability type (adjusted) odds ratios, 95% CI, p values** | | | | | | |
| --- | --- | --- | --- | --- | --- | --- |
|  |  |  |  |  |  |  |
| **Variables** | **Seeing** | **Hearing** | **Walking** | **Cognition** | **Self-care** | **Communicating** |
| **Disability** |  |  |  |  |  |  |
| Not Disabled | Ref | Ref | Ref | Ref | Ref | Ref |
| Disabled | 0.90[0.38 - 2.10] *0.802* | 1.31[0.16 - 10.46]*0.792* | 0.79[0.30 - 2.07]*0.614* | 1.03[0.52 - 2.06]*0.930* | 0.37[0.11 - 1.27] *0.129* | 0.39[0.05 - 3.13] 0.376 |
|  |  |  |  |  |  |  |
| **Age in Years** |  |  |  |  |  |  |
|  |  |  |  |  |  |  |
| 16 - 24 | Ref | Ref | Ref | Ref | Ref | Ref |
| 25 - 34 | 0.84[0.51 - 1.37] | 0.85[0.52 - 1.37] | 0.86[0.53 - 1.38] | 0.85[0.52 - 1.37] | 0.86[0.53 - 1.39] | 0.85[0.53 - 1.37] |
| 35 - 49 | 0.61[0.31 - 1.20], *0.172* | 0.60[0.31 - 1.17], *0.160* | 0.62[0.32 - 1.20], *0.183* | 0.60[0.31 - 1.18], *0.161* | 0.60[0.31 - 1.17], *0.163* | 0.61[0.32 - 1.18], *0.163* |
|  |  |  |  |  |  |  |
| **Gender** Male | Ref |  | Ref | Ref | Ref | Ref |
| Female | 0.51[0.36 - 0.74]*<0.001* | 0.52[0.36 - 0.75]*<0.001* | 0.52[0.36 - 0.74]*<0.001* | 0.52[0.36 - 0.75]*<0.001* | 0.51[0.36 - 0.74] *<0.001* | 0.51[0.35 - 0.73]*<0.001* |
|  |  |  |  |  |  |  |
| **Marital Status** |  |  |  |  |  |  |
| Singles | Ref | Ref | Ref | Ref | Ref | Ref |
| Paired | 0.30[0.18 - 0.52]*<0.001* | 0.30[0.18 - 0.53]*<0.001* | 0.29[0.17 - 0.50]*<0.001* | 0.31[0.18 - 0.53]*<0.001* | 0.30[0.18 - 0.52]*<0.001* | 0.30[0.18 - 0.52] *<0.001* |
|  |  |  |  |  |  |  |
| **No Poverty** |  |  |  |  |  |  |
| Very Poor | Ref | Ref | Ref | Ref | Ref | Ref |
| Moderately Poor | 0.97[0.62 - 1.53], *0.879* | 0.92[0.63 - 1.54], *0.922* | 0.98[0.63 - 1.52], *0.895* | 0.98[0.62 - 1.55], *0.923* | 0.97[0.62 - 1.53], *0.899* | 0.99[0.63 - 1.56] *0.952* |
|  |  |  |  |  |  |  |
| **Distance to HF (Km)** |  |  |  |  |  |  |
| 0 to 7km | Ref | Ref | Ref | Ref | Ref | Ref |
| Don’t know | 0.75[0.20 - 2.73] | 0.75[0.20 - 2.73] | 0.74[0.20 - 2.69] | 0.74[0.20 - 2.71] | 0.73[0.20 - 2.66] | 0.74[0.20 - 2.68] |
| 8 or more | 1.56[0.78 - 3.09], *0.194* | 1.60[0.82 - 3.13], *0.162* | 1.59[0.81 - 3.13], *0.168* | 1.60[0.81 - 3.15], *0.163* | 1.66[0.83 - 3.33], *0.141* | 1.62[0.82 - 3.22], *0.153* |
|  |  |  |  |  |  |  |
| **HIV Testing and Results** |  |  |  |  |  |  |
| Negative | Ref | Ref | Ref | Ref | Ref | Ref |
| Not tested | 0.63[0.38 - 1.06] | 0.63[0.38 - 1.06] | 0.64[0.38 - 1.06] | 0.63[0.37 - 1.06] | 0.65[0.39 - 1.08] | 0.63[0.38 - 1.06] |
| Positive | 4.33[0.96 - 19.56]*0.006* | 4.31[0.95 - 19.46] *0.006* | 4.37[0.96 - 19.80]*0.006* | 4.33[0.94 - 19.81]*0.006* | 4.35[0.96 - 19.66] *0.006* | 4.28[0.94 - 19.41] *0.006* |
|  |  |  |  |  |  |  |
| **SCT** No | Ref | Ref |  | Ref | Ref | Ref |
| Yes | 1.71[0.82 - 3.55] *0.126* | 1.54[0.62 - 3.82] *0.142* | 1.67[0.80 - 3.45] *0.143* | 1.68[0.80 - 3.51] *0.142* | 1.63[0.77 - 3.42]*0.165* | 1.65[0.79 - 3.43], *0.149* |
| N | 1139 | 1140 | 1140 | 1140 | 1140 | 1140 |
| Wald Test, Ref Reference, SCT social cash transfer, HF health facility, Km Kilometres. Disability was defined as answering 3 (“limited a lot”) or 4 (“cannot at all”) in performing the specific function. | | | | | | |
