# Supplementary material for: Differences in condom access and use and associated factors between persons with and without disabilities receiving social cash transfers in Luapula province, Zambia—A cross-sectional study
Source: PLoS One. 2024 Jun 6;19(6):e0302182. doi: 10.1371/journal.pone.0302182 (PMC11156379; doi:10.1371/journal.pone.0302182)
Supplement: S3 Table — (DOCX) [file pone.0302182.s003.docx]

| **S1 Table 3: Differences in factors associated with condoms access and use among 16 - 49 year olds, Odds Ratios, 95% CI** | | | | | |
| --- | --- | --- | --- | --- | --- |
|  | **Unadjusted (n=1143)** | ***p value*** |  | **Adjusted (n=1139)** | **p value** |
| **Difficulty level** |  |  |  |  |  |
| None | Ref |  |  | Ref |  |
| Mild | 0.69[0.45 - 1.06] | *0.088* |  | 0.80 [0.51 - 1.26] | *0.330* |
| Moderate | 0.85[0.49 - 1.48] | *0.565* |  | 1.07 [0.59 - 1.93] | *0.833* |
| Severe | 0.65[0.13 - 3.34] | *0.603* |  | 0.48 [0.08 - 2.76] | *0.339* |
|  |  |  |  |  |  |
| **Age in Years** |  |  |  |  |  |
| 16 – 24 | Ref |  |  | Ref |  |
| 25 – 34 | 0.76[0.48 - 1.22] | *0.263* |  | 0.85[0.52 - 1.398] | *0.523* |
| 35 – 49 | 0.38[0.22 - 0.64] | *<0.001* |  | 0.61[0.31 - 1.22] | *0.163* |
|  |  |  |  |  |  |
| **Sex** |  |  |  |  |  |
| Male | Ref |  |  | Ref |  |
| Female | 0.65[0.47 - 0.90] | *0.011* |  | 0.52[0.36 - 0.75] | *<0.001* |
|  |  |  |  |  |  |
| **Marital Status** |  |  |  |  |  |
| Single | Ref |  |  | Ref |  |
| Paired | 0.29[0.19 - 0.45] | *<0.001* |  | 0.30[0.17 - 0.52] | *<0.001* |
|  |  |  |  |  |  |
| **No Poverty** |  |  |  |  |  |
| Very Poor | Ref |  |  | Ref |  |
| Moderately Poor | 0.93[0.60 - 1.45] | *0.759* |  | 0.98[0.62 - 1.53] | *0.912* |
|  |  |  |  |  |  |
| **Distance to Health Facility (Kilometres)** | | | | | |
| 0 to 7km | Ref |  |  | Ref |  |
| Don’t know | 0.77[0.23 - 2.56] | *0.668* |  | 0.73[0.20 - 2.68] | *0.630* |
| 8 or more | 1.49[0.76 - 2.92] | *0.246* |  | 1.60[0.80 - 2.68] | *0.638* |
|  |  |  |  |  |  |
| **HIV Testing and Results** | | | | |  |
| Negative | Ref |  |  | Ref |  |
| Not tested | 1.00[0.61 - 1.65] | *0.998* |  | 1.59[0.95 - 2.66] | *0.076* |
| Positive | 3.05[0.66 - 14.06 |  |  | 6.90[1.46 - 32.7] | *0.076* |
|  |  |  |  |  |  |
| **Social Cash Transfer Access** | | | | |  |
| No | Ref |  |  | Ref |  |
| Yes | 1.82[0.90 - 3.69] | *0.093* |  | 1.71[0.83 - 3.54] | *0.146* |

Wald Test, Ref Reference. Severity of functional difficulties were coding 0 for no difficulty, 1 for some difficulty, 6 for a lot of difficulty and 36 for cannot do at all.
