# Supplementary material for: Differences in condom access and use and associated factors between persons with and without disabilities receiving social cash transfers in Luapula province, Zambia—A cross-sectional study
Source: PLoS One. 2024 Jun 6;19(6):e0302182. doi: 10.1371/journal.pone.0302182 (PMC11156379; doi:10.1371/journal.pone.0302182)
Supplement: S1 Annex — (DOCX) [file pone.0302182.s004.docx]

**S1 annex: The Washington group short set of questions on disability**

The questions ask about difficulties you may have doing certain activities because of a HEALTH PROBLEM.

1. Do you have difficulty seeing, even if wearing glasses?

a. No - no difficulty

b. Yes – some difficulty

c. Yes – a lot of difficulty

d. Cannot do at all

1. Do you have difficulty hearing, even if using a hearing aid?

a. No- no difficulty

b. Yes – some difficulty

c. Yes – a lot of difficulty

d. Cannot do at all

1. Do you have difficulty walking or climbing steps?

a. No- no difficulty

b. Yes – some difficulty

c. Yes – a lot of difficulty

d. Cannot do at all

1. Do you have difficulty remembering or concentrating?

a. No – no difficulty

b. Yes – some difficulty

c. Yes – a lot of difficulty

d. Cannot do at all

1. Do you have difficulty (with self-care such as) washing all over or dressing?

a. No – no difficulty

b. Yes – some difficulty

c. Yes – a lot of difficulty

d. Cannot do at all

1. 6. Using your usual (customary) language, do you have difficulty communicating, for example understanding or being understood?

a. No – no difficulty

b. Yes – some difficulty

c. Yes – a lot of difficulty

d. Cannot do at all
