# Supplementary material for: Differences in condom access and use and associated factors between persons with and without disabilities receiving social cash transfers in Luapula province, Zambia—A cross-sectional study
Source: PLoS One. 2024 Jun 6;19(6):e0302182. doi: 10.1371/journal.pone.0302182 (PMC11156379; doi:10.1371/journal.pone.0302182)
Supplement: S2 Annex — (DOCX) [file pone.0302182.s005.docx]

**S2 annex: Impact of social protection on access and use of HIV services: A quasi experiment study in two urban and two rural districts of Zambia**

**household questionnaire**

Questionnaire number | | | | | of | | | | |

ALWAYS FILL IN BASIC INFORMATION ( 1 – 12) BEFORE THE INTERVIEW

Section 1 : Social Demographic profile

1. Today’s date: | | |-| | |-| | | **(DD-MM-YY)**
2. Time start interview: | | |-| | | **(24 hr clock)**
3. Time end interview | | |-| | | **(24 hr clock)**
4. District: | | | | |
5. CWAC name and id: | | | | |
6. Village/locality name | | | | |
7. Respondent id code | | | | |
8. Gender of respondent (Male = 1; Female = 2) | |
9. Age (in years) | | |
10. Marital status | | (Married or living together =1, divorced or separated = 2 , Widowed = 3, Never married and never lived together = 4)
11. Highest level of education (No education = 0, Primary = 1, Secondary = 3, Adult literacy = 4, Higher = 5) | |
12. Main economic activity | | (Employment = 1, running non farm business = 2, farming, fishing, forestry = 3, piece work = 5, unpaid family worker = 6, full time student = 8, too old or young to work = 9)
13. Employment status | |

(self employed = 1, government employee = 2, private sector employee = 3, NGO employee = 4, Household employee = 5, others __________= 6 )

1. Numerator name and code | | |
2. Supervisor name and code | | |

(Supervisor, please sign next to name after checking the work)

Seek consent using the consent form:

**INFORMED CONSENT STATEMENT**

Hello. How are are you? My name is (Enumerator name), and I am working with researchers from Lusaka. We are conducting a survey of households in this district, and your household was chosen to be interviewed in this community.

I would like to ask you and some members of your household some questions about your household and community. We are asking you these questions to find out more about your household, your household’s economic activities, health and other measures of wellbeing. We hope that this information will eventually benefit the community by allowing us to understand the challenges that households like yours face, and how to mitigate them. I will keep everything that you tell me entirely private and confidential, and will not talk to other people about what you have said. This interview will take 45 minutes.

You do not need to talk to me if you do not want to. And if there is any question you do not want to answer, that will be fine. It is important you understand that the answers you give will in no way affect your status with respect to the Ministry of Community Development and Social Services or other service providers you interact with. If you have any problems, or if you feel uncomfortable answering any question, you should feel free to stop talking with me at any time. You can speak with people in the District Social Welfare Office in Town for more details or clarification of this study.

Will you please give me some time to speak with you?”

1. *Respondent consent: _______________________ signed = 1 finger stamped = 2, verbally consent = 3*
2. *Respondent did not consent ____________________ Reason _________________*

**House-hold Roster :** I would like to start the interview by asking about yourself and the usual members of the household – those who live in this household.

| **1** | **2** | **3** | **4** | **5** | **6** | **7** | **8** |
| --- | --- | --- | --- | --- | --- | --- | --- |
| **Personal id number** | Please give me the names of all persons who **usually** live with this household. Start with the head of the household and include visitors who have lived with the household for **six months or more.** Include usual members, who are away visiting, in hospital, at boarding schools or college or university, etc. [**First Name, Last Name**] | How old s………now? RECORD EXACT AGE IN COMPLETED YEARS  FOR THOSE AGED 5 YEARS AND ABOVE.  FOR THOSE 0-59 MONTHS OLD.RECCORD THE AGE IN MONTHS USE UNDER FIVE CLINIC CARD IF AVAILABLE.  (SPECIFY AGE CODE BELOW)   1. YEARS 2. MONTHS   AGE CODE | Is .... male or female?  MALE=1 FEMALE=2 | Is the biological mother of ….. alive?  **1 YES, MOTHER LIVES IN HOUSEHOLD=WRITE PID**  **YES, BUT MOTHER NOT IN HOUSEHOLD=88**  **NO, MOTHER IS DEAD=99**  **DON’T KNOW=7777>>Q11** | Is the biological father of ….. alive?  **YES, FATHER LIVES IN HOUSEHOLD=WRITE PID YES,**  **BUT FATHER NOT IN HOUSEHOLD=88**  **IF NO, FATHER IS DEAD=**  **DON’T KNOW=7777>>Q13** | Is….. currently attending school?**[INCLUDING THOSE IN COLLEGES AND UNIVERSITIES]**  YES, NURSERY/PRE- SCHOOL=1 **>>Q13**  YES, OTHER GRADES FULL TIME=2  YES, OTHER GRADES PART TIME=3  YES COMM. SCHOOL FULL TIME=4  YES CORRESPONDENCE=5 YES ADULT LITERACY CLASS=6 **>> Q13**  YES TERTIARY SCHOOL=7 OTHER SPECIFY=8  NO=9 **>> Q14** | What is the marital status of ……..?  **NEVER MARRIED=1 MARRIED=2 SEPARATED=3 DIVORCED=4 WIDOWED=5 CO-HABITING=6** |
|  |  |  |  |  |  |  |  |
|  |  |  |  |  |  |  |  |
|  |  |  |  |  |  |  |  |
|  |  |  |  |  |  |  |  |
|  |  |  |  |  |  |  |  |
|  |  |  |  |  |  |  |  |
|  |  |  |  |  |  |  |  |
|  |  |  |  |  |  |  |  |
|  |  |  |  |  |  |  |  |

During the time you were pregnant with ______, did you receive any food supplementation or vitamin or micronutrient supplementation from a health service provider? (Yes/No/I don’t know/refuse to answer)

When _____was born, was the birth attended by a skilled attendant (midwife, doctor)? (Yes/No/I don’t know/refuse to answer)

In the years between ______’s birth and their entry into primary school, did you participate in any parenting programme? Or receive home-visits from someone who discussed parenting? (Yes/No/I don’t know/refuse to answer)

Thinking back now to when ______ was very young, could you tell us if he/she:

      Registered at birth? (Yes/No/I don’t know/refuse to answer)

      Was breast fed? [If yes, for how many months?] (Yes/No/I don’t know/refuse to answer)

      Slept most or all nights under an insecticide treated bed net? ((Yes/No/I don’t know/refuse to answer)

In the years between ______’s birth and their entry into primary school, did he/she attend a creche or preschool? (Yes/No/I don’t know/refuse to answer)

 In the years between ______’s birth and their entry into primary school, did he/she receive any food supplementation/participate in a feeding programme? If yes, at what age? (Yes/No/I don’t know/refuse to answer)

In the years between ______’s birth and their entry into primary school, what was your households main source of drinking water? [Add locally relevant options] (Yes/No/I don’t know/refuse to answer)

In the years between ______’s birth and their entry into primary school, what was type pf sanitation facilities did your household have?

**Disability Status:** I will ask you certain questions regarding difficulties you or your household members (below 16 years old) may have performing certain activities because of a health problem: (Have the Household answer only for HH members below 16 years old)

|  | Seeing | hearing | mobility | mental | Self care | Communicating |
| --- | --- | --- | --- | --- | --- | --- |
| Personal Id number | Do(es) (you) …. have difficulty seeing, even if wearing glasses?  1 - no difficulty 2. Yes – some difficulty 3. Yes – a lot of difficulty 4. Cannot do at all | Do(es) (you)…. have difficulty hearing, even if using a hearing aid?  1 - no difficulty 2. Yes – some difficulty 3. Yes – a lot of difficulty 4. Cannot | Do(es) (you) … have difficulty walking or climbing steps?  1 - no difficulty 2. Yes – some difficulty 3. Yes – a lot of difficulty 4. Cannot | Do(es) you … have difficulty remembering or concentrating?  1 - no difficulty 2. Yes – some difficulty 3. Yes – a lot of difficulty 4. Cannot | Do(es) (you) …. have difficulty (with self-care such as) washing all over or dressing?  1 - no difficulty 2. Yes – some difficulty 3. Yes – a lot of difficulty 4. Cannot | Using (your) (his/her) usual (customary) language, do you have difficulty communicating, for example understanding or being understood?  1 - no difficulty 2. Yes – some difficulty 3. Yes – a lot of difficulty 4. Cannot |
|  |  |  |  |  |  |  |
|  |  |  |  |  |  |  |
|  |  |  |  |  |  |  |
|  |  |  |  |  |  |  |
|  |  |  |  |  |  |  |
|  |  |  |  |  |  |  |
|  |  |  |  |  |  |  |
|  |  |  |  |  |  |  |
|  |  |  |  |  |  |  |
|  |  |  |  |  |  |  |
|  |  |  |  |  |  |  |

**Household HIV Services Access and use:** I will ask you questions on HIV services access and use for your household members below 16 years old (Have the Household answer only for HH members below 16 years old. HH members older than 16 years should be interviewed separately for the questions. If two HH of the same sex, interview only the older member)

|  | Testing | Testing | Test result | Circumcision | Circumcision | TB | TB | TB | TB |
| --- | --- | --- | --- | --- | --- | --- | --- | --- | --- |
| Personal Id number | Has … (Have you) ever tested for HIV? (Yes = 1, No = 2, Don’t know = 3, Refused = 9) | Why (has) (have you) …. never been tested for HIV? (Check all the applies)  (Don’t know where to test = 1, Test kits not available = 2, Testing center too far = 3, Afraid others will know = 4, don’t need test/risk too low = 5, Afraid spouse/partner will know = 6, Don’t want to know have HIV = 7, Religious reasons = 8 Refused = 9) | Is … (Are you) currently taking ARVs, that is anti-retroviral infections? )  By currently, I mean that you may have missed some doses but you are still taking ARVs).  (Yes = 1, No = 2, Don’t know = 3, Not HIV positive = 4, refused = 9) | Is …. (Are you) circumcised?  Skip this and the next question for female.  (Yes = 1, No = 2, don’t know = 3, refuse = 9 ) Circumcision is the complete removal of the foreskin from the penis. I have picture to show you what a completely circumcised penis. | Who did the circumcision?  (Doctor, clinical Officer, Nurse = 1, Traditional = 2, Midwife = 3, Other = 4, don’t know = 5, refused = 9) | (Has **NAME**) (Have you) ever visited a tuberculosis or TB clinic for TB diagnosis or treatment?  YES = 1  NO = 2  DON’T KNOW = 8  REFUSED = 9 | Have you ever been told by a doctor, clinical officer or nurse that (**NAME**) had TB?  YES = 1  NO = 2  DON’T KNOW = 8  REFUSED = 9 | Was (**NAME**) ever treated for TB?  YES = 1  NO = 2  DON’T KNOW = 8  REFUSED = 9 | The last time (Name) **you were tre**ated for TB, did **you** complete at least 6 months of treatment?  YES = 1, NO, THE MEDICINE WAS STOPPED IN LESS THAN 6 MONTHS = 2, NO, STILL ON TREATMENT = 3 DON’T KNOW = 8 REFUSED = 9 |
|  |  |  |  |  |  |  |  |  |  |
|  |  |  |  |  |  |  |  |  |  |
|  |  |  |  |  |  |  |  |  |  |
|  |  |  |  |  |  |  |  |  |  |
|  |  |  |  |  |  |  |  |  |  |
|  |  |  |  |  |  |  |  |  |  |
|  |  |  |  |  |  |  |  |  |  |
|  |  |  |  |  |  |  |  |  |  |
|  |  |  |  |  |  |  |  |  |  |
|  |  |  |  |  |  |  |  |  |  |
|  |  |  |  |  |  |  |  |  |  |

***Household PMTCT.*** *I will ask you questions on Prevention of Mother to Child Transmission for your household members below 16 years old* (Have the Household answer only for HH members below 16 years old. HH members older than 16 years should be interviewed separately for the questions. If two HH of the same sex, interview only the older member)

|  | Testing in ANC | ARVs in PMTCT | Reasons not taking PMTCT |
| --- | --- | --- | --- |
| Personal identification | Were you tested for HIV during any of your antenatal care clinic visits when you were pregnant ?  (Yes = 1, No = 2, I don’t know = 3, Did not go for antenatal = 4, Refuse = 9 | Did you take ARVs during your pregnancy with **(NAME your child)** to stop from getting HIV?  (Yes = 1, No = 2, I don’t know = 3, Refuse = 9 ) | What was the main reason you did not take ARVs while you were pregnant ?  (Was not prescribed = 1, felt healthy/not sick = 2, Cost of Medication = 3, Cost of Transport = 4, Religious reasons = 5, Was taking traditional medicines = 6, Medications out of stock = 7, Did not want people to know HIV status = 8, refuse = 9 |
|  |  |  |  |
|  |  |  |  |

**Section 1: Health Status: I am going to ask you about your General health status**

1. Have you been sick or injured during the last two weeks? | |

(Yes sick=1 Yes injured=2 Q3 Yes both=3 No=4 >>Q25

1. What were you mainly suffering from? (check all that apply) | | | | | | | | | |

(Fever/Malaria=01 Cough/Cold/Chest infection=02 Tuberculosis (TB)= 03 Asthma=04 Bronchities/Pneumonia/Chest pain=05 Diarrhoea=06 Skin rash infection =07 Diabetes/sugar disease=8 HIV/AIDS=9 Mental Health =10 Alcoholism = 11 OTHER(SPECIFY)) =12

1. Did you consult any health or other institutions or person for this illness or injury? | || |

(Consulted = 1, self administered medicine only = 2, did nothing = 3)

1. How much in total did you spend on medication and consultation in the last week? | || || || | (Give amount in Kwacha including cash and in-kind)
2. Where did you get the medicines from? | |

(Government institution = 1, Mission institution = 2, Private institution = 3, Pharmacy/Chemist = 4, traditional healers = 5, friends, neighbours, relatives = 6, others _____________ (specify= 7))

1. What was the method used to pay for the services ? | |

(Prepayment scheme including insurance = 1, paid for by employer = 2, Paid part and other paid by employer, family, friends, scheme, insurance = 3, paid directly = 4, Paid = 5, didn’t pay = 6)

1. How would you rate your health? | |

(Excellent = 1, Very good = 2, Good = 3, Fair = 4, Poor = 5

**Section 1b: Health Status – Mental Health**

I am going to ask you about your mental health status

1. Have you felt restless, like you can’t keep still? (anxiety item) | |

(Yes = 1, No=2)

1. Have you felt very fearful (i.e. scared or afraid) (Anxiety item) | |

(Yes = 1, No=2)

1. Have you lost interest in things (That is things you usually enjoy?) depression item | |

(Yes = 1, No=2)

1. Have you felt very trapped or caught (e.g. like you are trapped in a situation you cannot get out of) depression item (Yes = 1, No=2) | |
2. Have you had a lot of trouble sleeping? Depression and trauma item | |

(Yes = 1, No=2)

1. Have you had a lot of pain in your body? Trauma item (Yes = 1, No=2) | |
2. Have you felt very worthless? (ie. You have no worth or value) (Yes = 1, No=2) | |
3. Do you ever have thoughts of hurting or killing yourself ? (suicide) (Yes = 1, No=2)| |
4. Which health or other institutions or persons did you visit for any of the mental health issues identified? ( Govt health facility = 1, mission institution = 2, traditional healer = 3, Faith/spiritual healer = 4, did not visit = 5, Others ________ friends, family, neighbour etc = 6) | |

**Section 2: Access to Social Economic Services and Facilities (For social inclusion – restriction in participation).**

I am going to ask you about your access to social economic services.

| **Social-Economic services** | | | | | | |
| --- | --- | --- | --- | --- | --- | --- |
|  | 1 | | 2 | 3A | 3B |  |
| Facility Code | Facility Type | Do you know where the nearest ….. is located?  YES. 1  NO….2 >> ***NEXT***  ***FACILITY*** | How far is it to the nearest………..?  **[*READ OUT FACILITIES*]**  **[*GIVE DISTANCE IN KM. IF LESS THAN A KILOMETRE ENTER 00 IF MORE THAN 90KM ENTER 90. IF DON’T KNOW ENTER 99*]** | Normally, by what means do you get there? Normally how long does it take you to get there using this means? | | |
|  |  |  |  | Normal means:  ON FOOT… 1  BICYCLE… 2  MOTORBIKE…..3 SCOTCH CART..4 PUBLIC TRANSPORT…..5  PERSONAL VEHICLE 6  OTHER (SPECIFY)…….7 | Normal time:  LESS THAN 10 MIN 1  BETWEEN10-19 MIN……2 BETWEEN20-29 MIN…….3 BETWEEN30- 59MIN……4  1 HOUR AND ABOVE …….5 | Are you limited in the amount of access to the social services indicted because of your impairment?  (Not limited = 1  (Yes fully limited = 2,  (Yes Partially limited = 3) |
|  | Food Market (super market) |  |  |  |  |  |
|  | Community Development and social Services |  |  |  |  |  |
|  | Primary school |  |  |  |  |  |
|  | High School |  |  |  |  |  |
|  | Secondary School |  |  |  |  |  |
|  | College and or tertiary |  |  |  |  |  |
|  | Employment Office |  |  |  |  |  |
|  | Health Facility (Health post/center/clinic/hospital) |  |  |  |  |  |
|  | Faith Based Institution (Church, Mosque, etc ) |  |  |  |  |  |
|  | Police station/post |  |  |  |  |  |
|  | Bank |  |  |  |  |  |
|  | Public transport (road, or rail, or water transport) |  |  |  |  |  |
|  | Disability Peoples Organization |  |  |  |  |  |
|  | Others Specify |  |  |  |  |  |

**Section 3: Access to Social Protection Programs**

I am going to ask you questions on access to social economic services.

|  | Government | | Non-Government | | Individuals not family members | |
| --- | --- | --- | --- | --- | --- | --- |
| Name of government program | During the past 12 months have you or any member of your household received money or goods, including food, clothing, livestock or medicines from any of the following government programs  YES…..1  N0…….2>>***Next Program*** | What is the total value of assistance received from this program in the **last 12 months**? [Convert in-kind assistance to Kwacha.]  KWACHA>>***Next***  ***Program*** | During **the past 12 months** have you or any member of your household received money or goods, including food, clothing, livestock or medicines from any NGO, church or other non- government group ?  YES……1 NO…….2>>***Q8*** | What is the total value of assistance received from all these non-government sources in the **last 12 months**? [Convert in-kind assistance to Kwacha.]  KWACHA | During the **past 12 months** has any member of your household received money or goods, including food, clothing, livestock or medicines from individuals who are not part of your household?  YES……1 NO…….2>>***Next Section*** | What is the total value of the aasistance received from all these non-household members in the last 12 months (Convert inkind assistance into Kwacha) |
| Farm Input Subsidy |  |  |  |  |  |  |
| Food Security Pack |  |  |  |  |  |  |
| PWAS |  |  |  |  |  |  |
| Disability Grant (Social Cash Transfer) |  |  |  |  |  |  |
| Scholarship |  |  |  |  |  |  |
| School Uniforms |  |  |  |  |  |  |
| Other specify 1 |  |  |  |  |  |  |
| Other specify 2 |  |  |  |  |  |  |
| Other specify 3 |  |  |  |  |  |  |
| Other specify 4 |  |  |  |  |  |  |

1. Do you consider your household to be non poor, moderately poor or very poor? | |

(Non Poor = 1, Moderately Poor = 2, Very poor = 3)

1. In the past four weeks, did you or any household member go to sleep at hungry because there was not enough food? | |

(No = 0, Rarely – once or twice = 1, Sometimes – 3 to 10 times = 2, Often > 10 times)

**Section 4: Sexual and Reproductive Health**

I am going to ask you questions on access to sexual and reproductive health services

1. If you want any of the family planning methods, would it be easy for you to get or use one? | |

(Yes = 1, No = 2, Don’t know = 3, refused = 9) If 2,3,9 >> 2 otherwise skip

(Sterilization = 1, Pill = 2, IUD/Coil = 3, Injection =4, Implant = 5, Condoms (male and female) = 6, natural = 7, withdrawal = 8, Refused = 9, Not having sex = 10 , Other _____ specify = 11)

1. Why would it not be easy for you to get/ use a family planning method? (check all that apply) | |

(Not available/too far = 1, Not convenient = 2, Costs too much = 3, Embarassed = 4, don’t know where to get the method = 5, Don’t know how to use the method = 6, Partner does not want = 7, Afraid partner will leave = 8, Refused = 9)

1. During the last 12 months, have you had an abnormal discharge from your vagina or experienced pelvic pain? | |

This may include an unusual smell, color, or texture. (yes = 1 no = 2, don’t know = 3, REFUSED = 9) skip if male

1. During the last 12 months, have you had an abnormal discharge from your penis? | | (yes = 1 no = 2, don’t know = 3, REFUSED = 9) skip if female
2. Did you get treatment for these problems? (yes = 1 no = 2, don’t know = 3, REFUSED = 9) | |
3. Where did you go to get treatment? Select all that apply | || || || || | |

(Government institution = 1, Mission institution = 2, Private institution = 3, Pharmacy/Chemist = 4, traditional healers = 5, friends, neighbours, relatives = 6, others _____________ (specify= 7))

1. Have you ever been tested for cervical cancer? (Skip for males) | |

(Yes = 1, No = 2, I don’t know = 3, Refused = 9)

1. What was the result of your last test for cervical cancer? | |

(Normal/negative = 1, abnormal = 2, suspect cancer = 3, inconclusive = 4, Did not receive results = 5, don’t know = 8, refused = 9

**Section 5 : Sexual and Gender based violence**

I am going to ask you questions on sexual and gender based violence.

1. Since age 15, has anyone other than your partner even forced you to have sex or perform a sexual act, when you did not want to, <? | |

YES = 1, NO = 2, DON’t know = 3, refused = 9

If yes : who did this to you ? Father, stepfather, other male family member, teacher, policy office, malre friend of he family, boyfriend, stranger, some at work, priest, other (check all applicable answers)

1. In the last 12 months, has your current or former partner ever?

- Punched, kicked, whipped, or beat you with an object | |
- Slapped you, threw something at you that could hurt you, pushed you or shoved you | |
- Choked smothered, tried to drown you, or burned you intentionally | |
- Used or or threatened you with a knife, gun or other weapon? | |
- Forced you to do something that you found degrading or humiliating

(YES = 1, nO = 2, don’t know =3, refused = 9)

1. In the last 12 months, did your partner physically force you to have sex? | |

(By partner, I mean a live-in partner whether or not you were married at the time). YES =1 NO, DID NOT FORCE = 2 NO, DID NOT HAVE A LIVE-IN PARTNER IN THE LAST 12 MONTHS= 3 DON’T KNOW = 8 REFUSED = 9)

1. After any of these unwanted sexual experiences, did you try to seek professional help or services from any of the following? SELECT ALL THAT APPLY. | || || || || || || | |

(Government institution = 1, Mission institution = 2, Private institution = 3, traditional healers = 4, friends, neighbours, relatives = 5, I did not seek help = 6, others _____________ (specify= 7)) If 6>>9

1. What was the main reason that you did not try to seek professional help or services? (Skip if sought services. Check all that applies) | || || || || || || | |

(Did not know services were available = 1, Services were not available = 2, Afraid of getting into trouble = 3, Ashamed of myself/family = 4, could not afford services = 5, Did not think it was a problem = 6, Felt it was my fault = 7, Afraid of being abandoned = 8, Did not need/want services = 9, Afraid of making situation worse = 10)

**Section 6: Access to HIV services**

Now I would like to ask you questions on HIV testing, treatment, prevention, care and support

1. Have you ever tested for HIV? (Yes = 1, No = 2, Don’t know = 3, Refused = 9) | |

If 2 go to 3, For 3 and 9 go to 6

1. Why have you never been tested for HIV? (Check all the applies) | || || || || || || | |

(Don’t know where to test = 1, Test kits not available = 2, Testing center too far = 3, Afraid others will know = 4, don’t need test/risk too low = 5, Afraid spouse/partner will know = 6, Don’t want to know have HIV = 7, Religious reasons = 8 Refused = 9)

1. What was the result of that (most recent) HIV test? | |

(Positive = 1, Negative = 2, Indeterminate = 3, did not receive result = 4, don’t know = 5)

1. Are you currently taking ARVs, that is anti-retroviral infections? ) | |

By currently, I mean that you may have missed some doses but you are still taking ARVs).

(Yes = 1, No = 2, Don’t know = 3, Not HIV positive = 4, refused = 9)

1. Are you circumcised? Skip this and the next questions for female. | |

For 2 go to next question (Yes = 1, No = 2, don’t know = 3, refuse = 9 ) Circumcision is the complete removal of the foreskin from the penis. I have picture to show you what a completely circumcised penis.

1. Who did the circumcision? | |

(Doctor, clinical Officer, Nurse = 1, Traditional = 2, Midwife = 3, Other = 4, don’t know = 5, refused = 9)

1. Were you tested for HIV during any of your antenatal care clinic visits when you were pregnant ? | |

(Yes = 1, No = 2, I don’t know = 3, Did not go for antenatal = 4, Refuse = 9 )

1. Did you take ARVs during your pregnancy with **(NAME your child)** to stop from getting HIV? | |

(Yes = 1, No = 2, I don’t know = 3, Refuse = 9 )

1. What was the main reason you did not take ARVs while you were pregnant ? | |

(Was not prescribed = 1, felt healthy/not sick = 2, Cost of Medication = 3, Cost of Transport = 4, Religious reasons = 5, Was taking traditional medicines = 6, Medications out of stock = 7, Did not want people to know HIV status = 8, refuse = 9

1. Have you ever visited a tuberculosis or TB clinic for TB diagnosis or treatment? | | YES = 1 ; NO = 2, DON’T KNOW = 8, REFUSED = 9
2. Have you ever been told by a doctor, clinical officer or nurse that you had TB?

| | YES = 1, NO = 2, DON’T KNOW = 8, REFUSED = 9

1. Were you ever treated for TB? | |

YES = 1, NO = 2, DON’T KNOW = 8, REFUSED = 9

1. The last time **you were tre**ated for TB, did **you** complete at least 6 months of treatment? | |

YES = 1, NO, THE MEDICINE WAS STOPPED IN LESS THAN 6 MONTHS = 2, NO, STILL ON TREATMENT = 3 DON’T KNOW = 8 REFUSED = 9

Section 7: Capacity Building to Access SRH HIV and Social Protection

Now I will ask you on the type and number of trainings you have received to increase you ability to access rights HIV, sexual and reproductive services and social protection

|  | Government provided | | Non-Government provided | | Not Government nor Non-Governmental Organisation | |
| --- | --- | --- | --- | --- | --- | --- |
| Name of Training | During the past 12 months have you or any member of your household received training and or any capacity building on the following topics provided by the goverment  YES…..1  N0…….2>>***Next Program*** | What is the total number of trainings received? (Number) | During the past 12 months have you or any member of your household received training and or any capacity building on the following topics provided by non-govermental organisations including Disability People’s Organisation and FaithBased organisations  YES…..1  N0…….2>>***Next Program*** | What is the total number of trainings received? (Number) | During the past 12 months have you or any member of your household received training and or any capacity building on the following topics provided by anyone else other than government and non-govermental organisations  YES…..1  N0…….2>>***Next Program*** | What is the total number of trainings received? (Number) |
| General Health Education |  |  |  |  |  |  |
| Mental Health |  |  |  |  |  |  |
| Education in including Adult literacy |  |  |  |  |  |  |
| Job Skills training |  |  |  |  |  |  |
| Economic Empowerment |  |  |  |  |  |  |
| Social Protection |  |  |  |  |  |  |
| Gender Based Violence |  |  |  |  |  |  |
| Human Rights |  |  |  |  |  |  |
| Sexual and Reproductive Health |  |  |  |  |  |  |
| HIV |  |  |  |  |  |  |
| Disability Sensitive planning |  |  |  |  |  |  |
