# Supplementary material for: Differences in condom access and use and associated factors between persons with and without disabilities receiving social cash transfers in Luapula province, Zambia—A cross-sectional study
Source: PLoS One. 2024 Jun 6;19(6):e0302182. doi: 10.1371/journal.pone.0302182 (PMC11156379; doi:10.1371/journal.pone.0302182)
Supplement: S3 Annex — (DOCX) [file pone.0302182.s006.docx]

**S3 annex: Impact of social protection on access and use of HIV services: A quasi experiment study in two urban and two rural districts of Zambia household questionnaire (Bemba)**

| Time Start Interview |
| --- |
| Device ID |
| Select Supervisor |
| Select Enumerator |
| Cluster ID |
| Household ID |
| District Name |
| PLEASE CONFIRM THE DETAILS  Full Name: ${pull_fname}, ${pull_lname} Gender: ${pull_gender} NRC Number: ${pull_nrc} CWAC: ${pull_cwac} WARD: ${pull_ward} Are these details correct? |
| What is the name of the village? |
| Ishina lyandi ninebo_____mfumine kukabungwe ka Palm Associates ku Lusaka mukubombela pamo nechiputulwa cha Community Development.  Tuli nomulimo wakufwailisha mumayanda aya lembeshiwa nangu ukwafwilishiwapo kwa balanda pamo na bantu abalemana ukufuma kuchiputulwa cha Community Development pamo nga Social Cash Transfer mwiboma lyenu. Ing'anda yenu naisalwa ukusendemo ulubali muli ukukufwailisha  muli uno mushi. Kuti natemwa ukwipusha imwe nabo mwikala nabo bonse abali nemyaka yakufyalwa ukuchila pekumi limo na mutanda amepusho ayakumine imikalile, imibombele, ubumi ukubikapofye nobwikashi bwenu. Tulechetekela amasuko yenu yakafwilisha saana umushi wenu munshila yakutila abo chikumine bakeshiba bwino bwino amafya yantu mukwete nefyo yanga bombelwapo. Uku kulanshanya kwankama kabili tatwakebe abantu bambi  pamasuko yenu. Mukwai, twalasendafye bamineti amakumi yasano muli ukukulanshya.  Mulibantungwa ukukanasendamo ulubali ngatamulefwaya. Kabili kuti mwakana ukwasuka ilipusho ilili lyonse, chilifye bwino. Nakuchilisha, kuti mwaleka ukulanda naifwe ishita ilifye yonse.  Chikankala mwishibe ukutila amasuko yenu tayakalete ubwafya nangu ubusanso mushila ililiyonse ukulola kumyumfwanine pakati kenu ne chiputulwa cha Ministry of Community Development ans Social Services. Ngamulefwaya ukwishilapo nafimbi pali uku kufwailisha kuti mwalanshanya na babomfi baku District Social Welfare Office mwiboma lyenu. Bushe muliko namepusho?  Mwasumina ukusendamo ulubali muli uku kulanshanya? |
| Do you agree to participate in the survey? |
| Let the respondent sign on the tablet |
| What was the reason for not giving consent? |
| Impact of Social Protection Programs |
| Impendwa yabekashi bapano pang'anda |
| Section B: Household Roster |
|  |
| Device ID |
| Calculated Household ID |
|  |
| Person ID |
| Member no. ${pid} full name |
| What is ${C1}'s Nick-Name? |
| What is ${C1}'s age in years? |
| What is ${C1}'s age in months? |
| What is ${C1}'s Gender? |
| Bushe umutwe wa ng'anda mulinabo shani? |
| Mulaya ku sukulu? |
| londololeni |
| What is ${C1}'s marital status? |
| Device ID |
| Bushe banina bakumufyala epo baba? |
| please specify the mother's PID |
| Bushe bawishi bakumufyala epo baba? |
| Please specify the father's PID |
|  |
| What is the phone number used at this household? |
| Household head age |
| Household head gender |
| Household head fullnmae |
| Time start general health status |
| Device ID |
| Calculated Persons ID |
|  |
| Time start general health status |
| Bushe mukumona kwenu pano pang'anda, kuti mwatila tamwachula, nangu mwalichulako panono, nangu mwalichula sanafye? |
| Mumilungu 4 iyapita, mushe mwalilalapo nangula abekashi bapano pang'anda balilalapo nensala pamulandu wakubuliwa ifyakulya? |
| Mwalilwalapo nangula ukuicena muli iyi milungu ibili iyapita? |
| Bwafya nshi mwaleshingwanabo sana sana? |
| londololeni |
| Bushe mwalifywailikishapo ubwafywilisho ukufuma ku chipatala nangula kukabungwe akalikonse, atemwa kubantu abalibinse pali uku kulwala/ ukuicena? |
| Mwaposelepo shinga pabundapisha, muli uyu mulungu wapita? |
| Bushe mwafumishe kwisai umuti? |
| londololeni |
| Bushe mwalipilile mumusangonshi? |
| Bushe mukumona kwenu ubumi bwenu bwaba shani? |
| Bushe mwalyumfwako ukubensauka mumitontonkanishishe,ichakwebati mwafilwa nokushikatala/ukwikalikana? |
| Bushe mwalyumfwapo ukupopomenwa, umwenso nangula ukutina? |
| Bushe mwalyumfwapo ukutompokwa mufintu ifimyumfwisha bwino ilingiline? |
| Bushe mwalyumfwapo ukukakwa nangula ukulubila mwilangulushi ichakwebati mulefilwa nokutontonkanya pafintu fimbi? |
| Bushe mulashupikwako notulo? |
| Bushe mwalikweteko ukukalipwa saana kwamubili? |
| Bushe mwaliyufwapo ukukanaba uwachindama? |
| Bushe mwalikwatapo amatontonkanyo ayakuipaya nangula ukuicena? |
| Nikwisa mwaile mukwafwilishiwa pali aya amafya mwalumbula? |
| londololeni |
|  |
| Bushe mwalishiba uko mwingasanga maliketi/amatuka yafyakulya mupepi? |
| yabela ukutali shani? |
| Mwenda shani pakuya uku? |
| Londololeni |
| Chisenda inshita iitali shani ukufika? |
| Bushe mwaliba aba pelebelwa kukuya ku maliketi kumulandu wabulema bwenu? |
| Bushe mwalishiba ukwabela amaofeshi ya community development and social services |
| yabela ukutali shani? |
| Mwenda shani pakuya uku? |
| Londololeni |
| Chisenda inshita iitali shani ukufika? |
| Bushe mwaliba aba pelebelwa kukuya ku maofeshi aya kumulandu wabulema bwenu? |
| Bushe mwlishiba ukwabela isukulu lya pelela pa grade seven? |
| yabela ukutali shani? |
| Mwenda shani pakuya uku? |
| Londololeni |
| Chisenda inshita iitali shani ukufika? |
| Bushe mwaliba aba pelebelwa kukuya kuli ili isukulu kumulandu wabulema bwenu? |
| Bushe mwalishiba ukwabela isukuli lyaambila pa grade 10 ukupelela pa grade 12? |
| Bushe lyabela ukutali shani? |
| Mwenda shani pakuya uku? |
| Londololeni |
| Bushi chisenda inshita iitali shani ukufika? |
| Bushe mwaliba abapelebelwa mukwenda ukufika kwisukulu ili pamulandu wabulema bwenu? |
| Bushe mwalishiba ukwabela isukuli lyaambila pa grade 8 ukupelela pa grade 9? |
| Bushe lyabela ukutali shani? |
| Mwenda shani pakuya uku? |
| Londololeni |
| Bushi chisenda inshita iitali shani ukufika? |
| Bushe mwaliba abapelebelwa mukwenda ukufika kwisukulu ili pamulandu wabulema bwenu? |
| Bushe mwalishiba ukwabela isukuli lyamasambililo yapamulu? |
| Bushe lyabela ukutali shani? |
| Mwenda shani pakuya uku? |
| Londololeni |
| Bushi chisenda inshita iitali shani ukufika? |
| Bushe mwaliba abapelebelwa mukwenda ukufika kwisukulu ili pamulandu wabulema bwenu? |
| Bushe mwalishiba ukwabela iofeshi lyakwingiliko inchito mupepi? |
| Bushe lyabela ukatali shani? |
| Mwenda shani pakuya uku? |
| Londololeni |
| Bushe chisenda inshita iitali shani ukufika? |
| Bushe mwaliba abapelebelwa mukwenda ukufika kuli ili iofeshi pamulandu wabulema bwenu? |
| Bushe mwalishiba ukwabela icikulwa cabundapishi(ichipatala, clinic, health post, centre)? |
| Bushe cabela ukutali shani? |
| Mwenda shani pakuya uku? |
| Londololeni |
| Bushe cisenda inshita iitali shani ukufika? |
| Bushe mwaliba abapelelwa mukwenda ukufika uku kumalandu wabulema bwenu? |
| Bushe mwalishiba ukwabela ichilonganino ca mapepo mupepi? |
| Bushe cabela ukutali shani? |
| Mwenda shani pakuya uku? |
| Londololeni |
| Bushe cisenda inshita iitali shani ukufika? |
| Bushe mwaliba abapelebelwa mukwenda ukufika ku ichilonganino kumulandu wabulema bwenu |
| Bushe mwlishiba ukwabela iofeshi lybakapokola mupepi? |
| Bushe lyabela ukutali shani? |
| Mwenda shani pakuya uku? |
| Londololeni |
| Chisenda inshita iitali shani ukufika? |
| Bushe mwaliba abapelebelwa mukwenda ukufika ku iofeshi lybakapokola kumulandu wabulema bwenu? |
| Bushe mwalishiba ukwabela ibanki lyandalama mupepi? |
| Bushe lyabela ukutali shani? |
| Bushe mwenda shani ukufika? |
| Londololeni |
| Bushe cisenda inshita iitali shani ukufika? |
| Bushe mwaliba abapelebelwa mukwenda ukufika ku ibanki lyandalama kumulanda wabulema bwenu? |
| Bushe mwalisha ukwbela inshila shakwendelamo isha chintu bwingi mupepi? |
| Bushe nikwisa shisangwa ishi nshila? |
| Bushe mwenda shani ukufika? |
| Londololeni |
| Bushe cisenda inshita iitali shani ukufika? |
| Bushe mwaliba abapelebelwa mukwenda ukufika ku ishila shakwendalamo kumulandu wabulema bwenu? |
| Bushe mwalishiba ukwabela akabungwe kabalema mupepi? |
| Bushe kabela ukutali shani? |
| Mwenda shani pakuya uku? |
| Londololeni |
| Bushe cisenda inshita iitali shani ukufika? |
| Bushe mwaliba abapelebelwa mukwenda ukufika kuli akabungwe kabalema pamulandu wabulema bwenu? |
| ACCESS TO SOCIAL PROTECTION PROGRAMS  Mumyenshi ikumilimo nashibili ishapita, bushe imwe nangu bambi pano ng'anda balipokapo ulupiya nangu ifintu ukubikapofye ifyakulya, ifyakufwala, ifitekwa, nangu umuti ukufuma kuma program yabuteko pamo nga? |
| Umufundo bapela mutubungwe twa chima? (in cooperatives) |
| Nishinga indalama shonse pamo ishinga poswapo pali uyumufundo mwapokele mumyenshi ikumilimo nashibili isha pita? (Convert in-kind assistance to estimated Kwacha) |
| Ukulya ifyakulya ku sukulu |
| Nishinga indalama shonse pamo ishinga poswapo pafyakulya balile abana benu ku sukulu mumyenshi ikumilimo nashibili isha pita? (Convert in-kind assistance to estimated Kwacha) |
| Ifyakulya ifipelwa mumayanda ayashikwanisha imilile bwino bwino (Food security pack) |
| Nishinga indalama shonse pamo ishinga poswapo pali ifyakulya mwapokele mumyenshi ikumilimo nashibili isha pita? (Convert in-kind assistance to estimated Kwacha) |
| Ifintu pamonga fyakufwala, insapato, amalangeti filyabapela ukufuma kuchiputulwa cha social welfare |
| Nishinga indalama shonse pamo ishinga poswapo pafintu mwapokele mumyenshi ikumilimo nashibili isha pita? (Convert in-kind assistance to estimated Kwacha) |
| Ulupiya lwabantu abalemana (Social cash transfer) |
| Nishinga indalama shonse pamo mwapokele mumyenshi ikumilimo nashibili isha pita? |
| Ubwafwilisho bwakulipilila abantu abali pamasambililo |
| Nishinga indalama shonse pamo bamilipilelipo ukutungilila amasambililo yenu nangu ayabambi pano ng'anda mumyenshi ikumilimo nashibili isha pita? (Convert in-kind assistance to estimated Kwacha) |
| Amauniform yaku sukulu |
| Nishinga indalama shonse pamo ishinga poswapo pamauniform yaku sukulu mwapokele mumyenshi ikumilimo nashibili isha pita? (Convert in-kind assistance to estimated Kwacha) |
| Fimbi 1 |
| Londololeni |
| Nishinga indalama shonse pamo ishinga poswapo pali fimbi(1) mwapokele mumyenshi ikumilimo nashibili isha pita? (Convert in-kind assistance to estimated Kwacha) |
| Fimbi 2 |
| Londololeni |
| Nishinga indalama shonse pamo ishinga poswapo pali fimbi(2) mwapokele mumyenshi ikumilimo nashibili isha pita? (Convert in-kind assistance to estimated Kwacha) |
| Fimbi 3 |
| Londololeni |
| Nishinga indalama shonse pamo ishinga poswapo pali fimbi(3) mwapokele mumyenshi ikumilimo nashibili isha pita? (Convert in-kind assistance to estimated Kwacha) |
| Fimbi 4 |
| Londololeni |
| Nishinga indalama shonse pamo ishinga poswapo pali fimbi(4) mwapokele mumyenshi ikumilimo nashibili isha pita? (Convert in-kind assistance to estimated Kwacha) |
|  |
| Mumyenshi ikumilimo nashibili ishapita, bushe imwe nangu bambi pano ng'anda balipokapo ulupiya nangu ifintu ukubikapofye ifyakulya, ifyakufwala, ifitekwa, nangu umuti ukufuma kukabungwe akashili kabuteko, chilonganino chamipepele (church) nangu ukufuma kutubungwe tumbi utu ibelele? |
| Nishinga indalama shonse pamo ishinga poswapo pali ububwafwilisho mwapokele mumyenshi ikumilimo nashibili isha pita ukufuma kutubungwe tonse utushili twabuteko? (Convert in-kind assistance to estimated Kwacha) |
| Mumyenshi ikumilimo nashibili ishapita, bushe imwe nangu bambi pano ng'anda balipokapo ulupiya nangu ifintu ukubikapofye ifyakulya, ifyakufwala, ifitekwa, nangu umuti ukufuma ku muntu umbi ushiliwapano ng'anda? |
| Nishinga indalama shonse pamo ishinga poswapo pali ububwafwilisho mwapokele mumyenshi ikumilimo nashibili isha pita ukufuma ku muntu umbi ushiliwapano ng'anda? (Convert in-kind assistance to estimated Kwacha) |
|  |
|  |
| CAPACITY BUILDING TO ACCESS SRH HIV AND SOCIAL PPROTECTION  Mumyenshi ikumi limo na shibili shapita bushe imwe nangu bambi pano nganda bali pokelelako amasambililo ya ibela nangu ukukanshiwa ukuchitwa nobuteko pamo nga |
| Amasambilio yafya bumi |
| Mwasendelemo ulubali imiku inga? |
| Amasambilio yamitontonkanishishe na kampingu |
| Mwasendelemo ulubali imiku inga? |
| Amasambilio yaba kalamba ukubikakofye nashibukeni |
| Mwasendelemo ulubali imiku inga? |
| Amasambilio yakukanshiwa palwamibombele |
| Mwasendelemo ulubali imiku inga? |
| Amasambilio yakutumpulula ubunonshi nechuma |
| Mwasendelemo ulubali imiku inga? |
| Amasambilio yapafyakulya nobu nonshi bwafiko ku bumi |
| Mwasendelemo ulubali imiku inga? |
| Amasambilo palwa kutungilila imikalile isuma (social protection) |
| Mwasendelemo ulubali imiku inga? |
| Amasambilo palwa ukuumana nokumana pakati kabashibantu naba na bantu |
| Mwasendelemo ulubali imiku inga? |
| Amasambililo yakumine insambu shabantu |
| Mwasendelemo ulubali imiku inga? |
| Amasambilio yabu shichifyashi nabunachifyashi |
| Mwasendelemo ulubali imiku inga? |
| Amasambilio palwa kashishi shika HIV |
| Mwasendelemo ulubali imiku inga? |
| Amasambililo yamyanshikile yamibombele isanshamo abalemana |
| Mwasendelemo ulubali imiku inga? |
| Mumyenshi ikumi limo na shibili shapita bushe imwe nangu bambi pano nganda bali pokelelako amasambililo ya ibela nangu ukukanshiwa ukuchitwa notubungwe utushili twabuteko (NGO) ukubikapofye utubungwe twabantu abalemana na utunbungwe twa filonganino (churches) |
| Amasambilio yafya bumi |
| Mwasendelemo ulubali imiku inga? |
| Amasambilio yamitontonkanishishe na kampingu |
| Mwasendelemo ulubali imiku inga? |
| Amasambilio yaba kalamba ukubikakofye nashibukeni |
| Mwasendelemo ulubali imiku inga? |
| Amasambilio yakukanshiwa palwamibombele |
| Mwasendelemo ulubali imiku inga? |
| Amasambilio yakutumpulula ubunonshi nechuma |
| Mwasendelemo ulubali imiku inga? |
| Amasambilio yapafyakulya nobu nonshi bwafiko ku bumi |
| Mwasendelemo ulubali imiku inga? |
| Amasambilo palwa kutungilila imikalile isuma (social protection) |
| Mwasendelemo ulubali imiku inga? |
| Amasambilo palwa ukuumana nokumana pakati kabashibantu naba na bantu |
| Mwasendelemo ulubali imiku inga? |
| Amasambililo yakumine insambu shabantu |
| Mwasendelemo ulubali imiku inga? |
| Amasambilio yabu shichifyashi nabunachifyashi |
| Mwasendelemo ulubali imiku inga? |
| Amasambilio palwa kashishi ka HIV |
| Mwasendelemo ulubali imiku inga? |
| Amasambililo yamyanshikile yamibombele isanshamo abalemana |
| Mwasendelemo ulubali imiku inga? |
| Mumyenshi ikumi limo na shibili shapita bushe imwe nangu bambi pano nganda bali pokelelako amasambililo ya ibela nangu ukukanshiwa ukuchitwa notubungwe nangula abantu abalibonse (ukufumishako ubuteko notubungwe utushili twabuteko) pamon nga... |
| Amasambilio yafya bumi |
| Mwasendelemo ulubali imiku inga? |
| Amasambilio yamitontonkanishishe na kampingu |
| Mwasendelemo ulubali imiku inga? |
| Amasambilio yaba kalamba ukubikakofye nashibukeni |
| Mwasendelemo ulubali imiku inga? |
| Amasambilio yakukanshiwa palwamibombele |
| Mwasendelemo ulubali imiku inga? |
| Amasambilio yakutumpulula ubunonshi nechuma |
| Mwasendelemo ulubali imiku inga? |
| Amasambilio yapafyakulya nobu nonshi bwafiko ku bumi |
| Mwasendelemo ulubali imiku inga? |
| Amasambilo palwa kutungilila imikalile isuma (social protection) |
| Mwasendelemo ulubali imiku inga? |
| Amasambilo palwa ukuumana nokumana pakati kabashibantu naba na bantu |
| Mwasendelemo ulubali imiku inga? |
| Amasambililo yakumine insambu shabantu |
| Mwasendelemo ulubali imiku inga? |
| Amasambilio yabu shichifyashi nabunachifyashi |
| Mwasendelemo ulubali imiku inga? |
| Amasambilio palwa kashishi shika HIV |
| Mwasendelemo ulubali imiku inga? |
| Amasambililo yamyanshikile yamibombele isanshamo abalemana |
| Mwasendelemo ulubali imiku inga? |
| Common Sections |
| Person ID |
| Calculated Household ID |
| Device ID |
| Persons Calculated Age in years |
|  |
| Persons Calculated Name |
| Pali ichi chipande afwile ukuyasukila umwine ngali ne myaka ikumi na mutanda? |
| INFORMED CONSENT for ${cfullname}   Ishina lyandi ninebo_____mfumine kukabungwe ka Palm Associates ku Lusaka. Mukubombela pamo nechiputulwa cha Community Development.  Tuli nomulime wakufwailisha mumayanda aya lembeshiwa nangu ukwafwilishiwapo kwa balanda pamo na bantu abalemana ukufuma kuchiputulwa cha Community Development pamo nga Social Cash Transfer mwiboma lyenu. Ing'anda yenu naisalwa ukusendemo ulubali muli ukukufwailisha  muli uno mushi kuti natemwa ukwipusha imwe nabo mwikala nabo bonse abali nemyaka yakufalwa ukuchila pekumi na mutanda amepusho ayakumine imikalile, imibombele, mbumi ukubikapofye nobwikashi bwenu. Tulechetekela amasuko yenu yakafwilisha. Saana umushi wenu munshila yakutila abo chikumine bakeshiba bwino bwino amafya yantu mukwebe nefyo yanga bombelwapo. Uku kulanshanya kwankama kabili tatwakebe abantu bambi  pamasuko yenu. Mukwai, twatasendafyw bamineti amakumi yasano muli ukukulanshya.  Mulibambungwa ukukanasendamo ulubali ngatamulefwaya. Kabili kuti mwakana ukwasuka ilipusho ilili lyonse, chilifye bwini. Nakuchilisha, kuti mwaleka ukulanda naifwe ishita ilifye yonse.  Chikankala mwishibe ukutila amasuko yenu tayakalete ubwafya nangu ubusamso mushila ililiyonse ukulola kumyumfwanine pakati kenu ne choputulwa cha Ministry of Community Development ans Social Services. Ngamulefwaya ukwishilapo nafimbi pali uku kufwailisha kuti mwalanshanya na babomfi baku District Social Welfare Office mwiboma lyenu. Bushe muliko namepusho?  Mwasumina ukusendamo ulubali muli uki kulanshanya? |
| Let the respondent sign on the tablet |
| What was the reason for not giving consent? |
| Second consent group |
| Time start Sexual and Reproductive Health |
| SEXUAL AND REPRODUCTIVE HEALTH   Enumerator: If the respondent wants any of the following family planning methods, would it be easy to get or use one? |
| Ngacakutila mulefwaya ukuputula / ukukaka inshila yabufyashi (sterlisasation), kuti caba icayangauka ukusanga nangu ukubomfya? |
| Ninshi cingabela icashupa ukusanga nangu ukubomfya ukuputula / ukukaka inshila yabufyashi (sterlisation)? |
| Ngacakutila mulefwaya ama pill, kuti caba icayanguka ukusanga nangu ukubomfya? |
| Ninshi cingabela icashupa ukusanga nangu ukubomfya ama pill? |
| Ngacakutila mulefwaya IUD/Coil , kuti caba icayanguka ukusanga nangu ukubomfya? |
| Ninshi cingabela icashupa ukusanga nangu ukubomfya IUD/Coil? |
| Ngacakutila mulefwaya inyeleti , kuti caba icayangukaukusanga nangu ukubomfya? |
| Ninshi cingabela icashupa ukusanga nangu ukubomfya inyeleti? |
| Ngacakutila mulefwaya iyakubika pakuboko (implant) , kuti caba icayanguka ukusanga nangu ukubomfya? |
| Ninshi cingabela icashupa ukusanga nangu ukubomfya iyakubika pakuboko (implant)? |
| Ngacakutila mulefwaya umupila (bashitata na banamayo) , kuti caba icayanguka ukusanga nangu ukubomfya? |
| Ninshi cingabela icashupa ukusanga nangu ukubomfya umupila (bashitata na banamayo)? |
| Ngacakutila mulefwaya ukubomfya iyakupenda inshinku (natural methods) , kuti caba icayanguka ukusanga nangu ukubomfya? |
| Ninshi cingabela icashupa ukusanga nangu ukubomfya iyakupenda inshinku (natural methods)? |
| Ngacakutila mulefwaya ukubomfya inshila yakuposa panse (withdrawal method) , kuti caba icayanguka ukusanga nangu ukubomfya? |
| Ninshi cingabela icashupa ukusanga nangu ukubomfya inshila yakuposa panse (withdrawal method)? |
| Ngacakutila mulefwaya ukukana kumana nabenamyenu , kuti caba icayanguka ukusanga nangu ukubomfya? |
| Ninshi cingabela icashupa ukusanga nangu ukubomfya inshila yakukana kumana nabenamyenu? |
| Ngacakutila mulefwaya inshila imbi iliyonse iyakucingilila ubufyashi , kuti caba icayanguka ukusanga nangu ukubomfya? |
| Ninshi cingabela icashupa ukusanga nangu ukubomfya inshila imbi? |
| Ninshilanshi imbi |
| Bushe ${cfullname} alikwatako ubwafya bwakumona nangu elyo afwele ama glasses? |
| Bushe ${cfullname} alikwata ubwafya bwakumfwa nangu nafwala/ alebomfya utwakumwafwili shako? |
| Bushe ${cfullname} alikwata ubwafya bwakwenda nangu ukunina pama steps? |
| Bushe ${cfullname} alikwatako ukushupikwa mukwibukisha nangula ukubikako amano? |
| Bushe ${cfullname} alikwatako ubwafya mukuisakamana, pamo nga ukuwasha,nemifwalile? |
| Bushe ${cfullname} alikwatako ubwafya ukumfwana nabambi muchitundu mubomfya ilingiline pakutila engomfwa nangula ukumumfwa? |
| Section C: Services Access and Use  ENUMERATOR: Please call ${cfullname} to respond to this section since they are over 16 years old |
| Bushe mwalitala amupimishapo akashishi ka HIV? |
| Bamisangile shani? |
| Cinshi calenga ukukana pimishapo akashishi ka HIV? |
| Bushe ${cfullname} palindakai mulanwa umuti wama ARVs? |
| People sometimes forget to take all their ARVs everyday. In the last 30 days, how many days have you missed taking any of your ARV pills? |
| Bushe${cfullname} mwalisembululwa? |
| Nibani bamisembulwile? |
| Bushe ${cfullname} mwaliyapo ku chipatala mukupimwa nangula ukundapwa kubulwele bwa chifuba chantanda bwanga (T.B)? |
| Bushe ${cfullname} balitala abamyebap kuli ba Doctor,Nurse, Clinical Officer ukuti mwalikwata ichifuba chantanda bwanga (T.B)? |
| Bushe ${cfullname} mwalitala ukundapwapo kubulwele bwa chifuba cha ntanda bwanga? |
| Umuka wakulekelesha ${cfullname} bamyundepe ichifuba ca ntandabwanga, bushe mwalikwanishe ukupoka ubundapishi ukufika imyeshi mutanda? |
| Section D: Prevention Of Mother to Child Transmission |
| Bushe ${cfullname} mwalipimishepo akashishi ka HIV, lintu mwaleya ku cipimo elyo mwali pabukulu? |
| Bushe ${cfullname} Mwalenwa umuti (ARVs) elyo mwali pa bukulu, pakuchingilila umwana ulimwifumo ukwambula akashIshi ka HIV? |
| Cinshi calengele ukutila mwilanwa umuti wama ARVs, elyo mwali pabukulu? |
|  |
|  |
| Time Start Specific Health Problmes |
| Mumyeshi 12 iyapita mwalitala kwatapo ifya kufuma kubwanakashi ifishilingile nangu ukukalipa muntungu? |
| Mumyeshi 12 iyapita mwalitala kwatapo ifya kufuma kubwaume ifishilingile? |
| Bushe mwalyundepwe pamafya aya? |
| Nikwisa uko bamyundapile? |
| Londololeni |
| Bushe mwalipimishapo Cancer isangwa kumulomo wacisa? |
| Bushe yafumine shani? |
|  |
| SGBV  Respondent: ${cfullname} |
| Section K: Sexual And Gender-based Violence |
| Ukutulafye lintu mwakwete imyaka ikumilimo nashisano, kwali bako abalibonse ukufumishako abena myenu abamipatikishe ukukumana nabo nangula ukumikata ikata pamubili ngati ilyo tamwalefwaya |
| Nibani bachitile ifi kuli imwe? |
| Londololeni |
| Mumyenshi ikumilimo nashibili ishapita, bushe abenamyenu nangu abatemwikwa benu abakale bali talile myumapo amakofi, ukumipanta, ukumilopola, nangu ukumyuma nechintu ichilichonse? |
| Mumyenshi ikumilimo nashibili ishapita, bushe abenamyenu nangu abatemwikwa benu abakale bali talile mitobapo amapi, ukumisunka? |
| Mumyenshi ikumilimo nashibili ishapita, bushe abenamyenu nangu abatemwikwa benu abakale bali talile mikamapo pamukoshi, ukumubike ichintu pamenso, ukuminwinsha nangu ukumyocha kumumbo? |
| Mumyenshi ikumilimo nashibili ishapita, bushe abenamyenu nangu abatemwikwa benu abakale bali talile bomfyapo nangu ukumitinya nomwele, infunti, nangu ichanso chimbi? |
| Mumyenshi ikumilimo nashibili ishapita, bushe abenamyenu nangu abatemwikwa benu abakale bali talile bomfyapo amaka ukumipatishika ukulungana naimwe lintu tamwalefwaya? |
| Mumyenshi ikumilimo nashibili ishapita, bushe abenamyenu nangu abatemwikwa benu abakale bali talile ulunganapo naimwe lintu talamwefwaya pantu mwalitinine ifyo bengachita nga mwakana? |
| Panuma ya ifi mwalumbula, bushe mwali talile fwayako ubwafwilisho nangula ifinga kabilwa ukufuma ku.. |
| Londololeni |
| Mulandushi ukalamba uwalengele ukutila mukane fwaya ubafwilisho nangu ifinga kabilwa |
| Londololeni |
|  |
| End second consent group |
|  |
| Time End Interview |
| GPS Location |
| What is the response status of the interview? |
| Time End Interview |
